# Real-time monitoring of live mycobacteria with a microfluidic acoustic-Raman platform

**DOI:** 10.1038/s42003-020-0915-3

**Published:** 2020-05-14

**Authors:** Vincent O. Baron, Mingzhou Chen, Björn Hammarstrom, Robert J. H. Hammond, Peter Glynne-Jones, Stephen H. Gillespie, Kishan Dholakia

**Affiliations:** 1 School of Medicine, University of St Andrews, KY16 9TF St Andrews, UK; 2 SUPA, School of Physics and Astronomy, University of St Andrews, KY16 9SS St Andrews, UK; 3 School of Engineering, University of Southampton, SO17 1BJ Southampton, UK; 4 Department of Physics, College of Science, Yonsei University, Seoul, 03722 South Korea

**Keywords:** Biophysics, Bacterial techniques and applications, Antibiotics, Applied microbiology

## Abstract

Tuberculosis (TB) remains a leading cause of death worldwide. Lipid rich, phenotypically antibiotic tolerant, bacteria are more resistant to antibiotics and may be responsible for relapse and the need for long-term TB treatment. We present a microfluidic system that acoustically traps live mycobacteria, *M. smegmatis*, a model organism for *M. tuberculosis*. We then perform optical analysis in the form of wavelength modulated Raman spectroscopy (WMRS) on the trapped *M. smegmatis* for up to eight hours, and also in the presence of isoniazid (INH). The Raman fingerprints of *M. smegmatis* exposed to INH change substantially in comparison to the unstressed condition. Our work provides a real-time assessment of the impact of INH on the increase of lipids in these mycobacteria, which could render the cells more tolerant to antibiotics. This microfluidic platform may be used to study any microorganism and to dynamically monitor its response to different conditions and stimuli.

## Abstract (web summary)

Baron et al. describe a microfluidic system that acoustically traps live mycobacteria and acquires label-free optical measurements over time using wavelength modulated Raman spectroscopy. Using acoustically trapped live *M. smegmatis*, they show that under stressed conditions produced by an antibiotic, bacteria displayed an increase in lipids which could render the cells more tolerant to antibiotics.

## Introduction

The complex changes in the composition of microorganisms are challenging to study continuously. Most methods involve repetitive sampling techniques that change the organisms or that are secondary measures of the primary target. Yet, small changes over small periods of time can be of importance to our understanding of the organism’s pathogenesis, its resistance profile, its ability to survive in a challenging environment or its interaction with other microorganisms. Thus, it would be beneficial to develop a method to capture, hold bacteria and to interrogate them by a method that did not, itself, change the bacteria.

Examples of current state of the art in technologies for real-time monitoring of bacterial populations include a fluorescent oxygen sensor in BACTEC MGIT^1^, electrical sensors^2^ and continuous measurement of the biomass^3^. However, these surrogate markers only give a limited view of changes in a complex cell content. Current microbiological methodologies used to study the character of bacteria usually require sequential sampling at specific time points. For example, sampling at given time points in the case of the hollow fibre allows some measure of insight into the pharmacokinetic effect of drugs^4,5^. Alternatively, sequential sampling can allow bacterial gene expression to be measured in a discontinuous way. This typically changes the culture, and is usually destructive. Consequently, it can provide only intermittent snapshots of variables that change continuously over time.

Acoustic trapping can produce and maintain suspended clusters of bacteria (recently demonstrated with *E. coli*^6^). This creates the possibility of monitoring a viable population of suspended bacteria over time, and to probe their response to stresses, including drugs and a changing environment. Compared with optical trapping, acoustic trapping can levitate cells over extended periods of many hours or weeks, with little heating or impact on cell viability^7,8^. It may be simply implemented using piezoelectric transducers, operating typically at megahertz frequencies. Importantly, a suitably designed trap can also facilitate real-time optical interrogation of the trapped bacteria. Raman spectroscopy offers a label-free, all-optical method to collect biochemical information from bacteria over time. In previous work, we have shown that wavelength modulated Raman (WMR) spectroscopy is a promising non-destructive methodology to study mycobacterial cell content for cells plated onto coverslips^9^. Raman measurements of small samples typically include a background signal created by the surface upon which they reside. A key advantage of the approach we describe here is the significant reduction in background signal achieved by holding the bacterial sample away from device surfaces with acoustic levitation. This creates a new platform for real-time interrogation of bacteria, and allows the biochemical effect of dynamic changes in nutrients and antibiotics to be studied.

Previously, standard Raman spectroscopy of acoustically trapped microparticles has been demonstrated^10^, and also of levitated droplets^11^. Infra-red spectroscopy of ultrasonically trapped particles in bioreactors has also been successful^12^. Acoustically trapping much smaller particles, such as bacteria in continuous flow systems, is challenging due to competition with acoustic streaming forces^13^. More recently, automatic sorting of isotopically labelled microbial cells was demonstrated with a combination of microfluidics, optical tweezers and Raman spectroscopy^14^.

In recent clinical trials, relapse was identified as a major barrier to shorten tuberculosis (TB) antibiotic treatment regimens^15–17^. There is increasing evidence supporting the clinical significance of the presence of lipid bodies in *M. tuberculosis* cells^18^. A higher risk of poor TB treatment outcome correlates with a higher proportion of mycobacteria with lipid inclusions in patients sputum after 3 and 4 weeks of treatment^19^. It is thought that lipid-rich mycobacteria with intracellular inclusions of non-polar lipids can be up to 40 times more resistant to first-line antibiotics compared with lipid-poor mycobacteria (those with an absence of intracellular inclusions of non-polar lipids)^20^. This phenomenon may play an important role in patients’ relapse^21^. It is important, therefore, to be able to study in real-time cell lipid concentrations under the influence of antibiotics, in vitro.

We present, to our knowledge, the first report of a platform that integrates optical Raman spectroscopy and acoustic trapping of such bacteria. To demonstrate its utility, we use the tool to study the effect of an antibiotic used in the standard TB regimen, isoniazid (INH), on a model organism (*M. smegmatis*) over an extended period. The results can aid detailed understanding of the impact of antibiotic-derived stress on the bacterial population, by recording quantitative and qualitative spectral changes occurring over time.

## Results

### No-stress condition

**Fig. 1:**
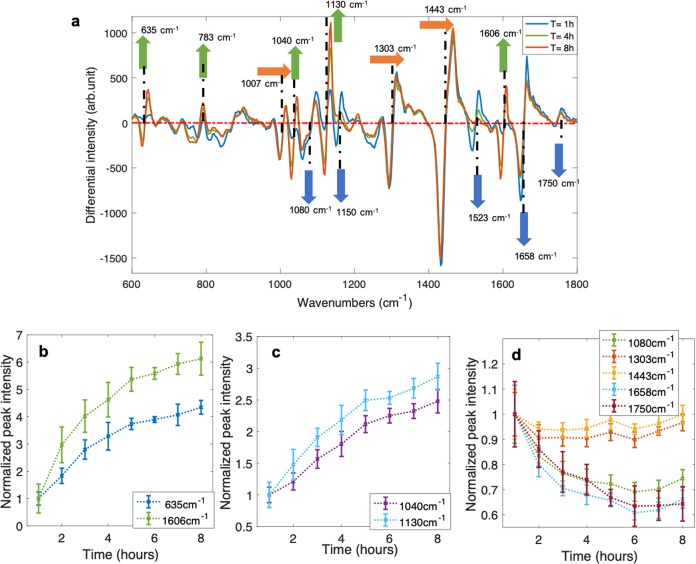
No-stress condition. **a** Shows the average WMR spectra for a no-stress experiment, presenting the first (T = 1 h; blue spectrum), an intermediate (T = 4 h, green spectrum) and the last (T = 8 h; red spectrum) time point of the experiment. The green, orange and blue arrows are, respectively, showing Raman peaks that are increasing, stable and decreasing over time based on the three no-stress experiments. The *x*-axis presents the wavenumbers in cm^−1^, while the *y*-axis shows the intensity in arbitrary unit. In WMR spectra, the zero crossings on the red dash-dotted line shows the positions of the Raman peaks. **b**–**d** Present the evolution of Raman peaks intensities over time. **b** Shows the Raman peaks at 635 cm^−1^ and 1606 cm^−1^ (associated with tyrosine) intensities during the experiment. **c** Presents the Raman peaks at 1040 cm^−1^ and 1130 cm^−1^ (associated with carbohydrates) intensities over time. **d** Shows the Raman peaks at 1080, 1303, 1443, 1658 and 1750 cm^−1^ (associated with lipids) intensities over the course of the experiment. The *x*-axis shows the time in hours, and *y*-axis the normalised intensity of the Raman peaks.

To understand the behaviour of organisms in this system, we examined their behaviour under normal growth conditions over time. In these "no-stress" experiments, a suspension of 7-day-old *M. smegmatis* was re-suspended in fresh 7H9 broth, and acoustically trapped in the microfluidic chamber. Bacteria were then measured using WMR spectroscopy for up to 8 h. Average Raman spectra, of one no-stress experiment, corresponding to the first (T = 1 h), fourth (T = 4 h) and eighth (T = 8 h) hour of the experiment are presented in the Fig. 1a. Average Raman spectra for all no-stress experiments are shown in Supplementary Fig. 2. The evolution of Raman peaks over time for all experiments are presented in Supplementary Figs. 3–8.

Among the Raman peaks investigated, several (635, 783, 1040, 1130 and 1606 cm^−1^) were increasing, several (1080, 1150, 1523, 1658 and 1750 cm^−1^) were decreasing and three (1007, 1303, 1443 cm^−1^) remained largely stable during the time-frame investigated. The most likely Raman peak assignments and their associated chemical bonds are shown in Table 1. When there are several potential assignments for one specific Raman peak (1080, 1130, 1443, 1606 and 1658 cm^−1^), the assignment associated with the increase or decrease of the Raman peak over time during the experiments is shown, when possible, in the no-stress or the INH-stress column between brackets.

**Table 1:** Raman peaks with their assignments and overall trends using three experiments for each condition over time (up to 8 h of measurement).

Raman peak (cm^−1^)	**Associated chemical bonds**	**Potential Raman peak assignments**	**No stress**	**INH stress**
635	C–C, C–S	Tyrosine^40–42^	Increasing	Increasing
783		Nucleic acids^41,42^	Increasing	Stable
1007	C–C	Phenylalanine^40–42^	Stable	Stable
1040		Carbohydrates^42^	Increasing	Increasing
1080	C–C, C–O–H, C–N, C–O	Lipids, carbohydrates, proteins^22,23,42,43^	decreasing	Increasing (lipids)
1130	C–N, C–C, C–O	Carbohydrates, proteins, lipids^40,42^	Increasing (carbohydrates)	Increasing (carbohydrates, possibly lipids)
1150	C–C	Carotenoids^41,42,44,45^	Decreasing	Decreasing
1303	CH_2_	Lipids^22,23,41–43^	Stable	Increasing
1443	CH_2_, CH_3_	Lipids, proteins ^22,23,40–43^	Stable	Increasing (lipids)
1523	C=C	Carotenoids^41,42,44,45^	Decreasing	Decreasing
1606	C=C	Tyrosine, phenylalanine^40–42^	Increasing (tyrosine)	Increasing (tyrosine)
1658	C=C, C=O	Lipids, proteins, amide I^22,40–43^	Decreasing	Increasing (lipids)
1750	C=O	Lipids^42,43,23^	Decreasing	Increasing (lipids)

The most likely assignment for the Raman peak at 635 cm^−1^ is tyrosine. Regarding the Raman peak located at 1606 cm^−1^, the assignment can be either phenylalanine or tyrosine. However, the Raman peak at 1007 cm^−1^, which is strongly associated with phenylalanine and not with tyrosine, was found to be stable throughout the 8 h of measurement (see Supplementary Fig. 7). In contrast, both 635 cm^−1^ and 1606 cm^−1^ represent the largest increase over the first 8 h of the experiment (see Fig. 1a, b). Raman peaks at 635 cm^−1^ and 1606 cm^−1^ may represent a rapid increase of tyrosine in trapped bacteria (see Fig. 1a, b).

The Raman peak at 1130 cm^−1^ can be assigned to several groups of molecules (lipids, proteins and carbohydrates). The Raman peak at 1443 cm^−1^ shows information on both lipids and proteins, and it is found stable over the first 8 h of the experiment. Both the Raman peaks located at 1040 cm^−1^ and 1130 cm^−1^, demonstrate a large increase over the first 8 h of the experiment. The increase in these two Raman peaks could be due to an increase in carbohydrate in trapped bacteria (see Fig. 1a, c).

The Raman peak at 783 cm^−1^, associated with nucleic acids, is increasing over time. This can be observed on the average spectra Fig. 1a.

Carotenoids, that can be observed in Raman peaks at 1150 cm^−1^ and 1523 cm^−1^, are rapidly reducing over time, suggesting that the organisms is using the stored material when placed in fresh medium. After only few hours in fresh medium, the carotenoid peaks have almost disappeared in the average spectra (Fig. 1a). We also observed a high variability in carotenoid associated Raman peak intensities from a clear high peak to a small intensity Raman peak, between the different experiments in Supplementary Fig. 6.

The intensity of Raman peaks at 1303 cm^−1^ and 1443 cm^−1^ remains largely unchanged over the 8 h of the measurement (see Fig. 1a). This suggests that both lipid and protein overall concentration in the trapped bacteria is not increasing or decreasing over time in no-stress experiments. The Raman peak at 1658 cm^−1^, associated with C=C or C=O, is slightly reducing over time (see Fig. 1a, d). The Raman peaks at 1080 cm^−1^ and 1750 cm^−1^ are both either stable or slightly reducing over time. In addition, the increase in Raman peaks at 1040 cm^−1^ and 1130 cm^−1^ due to carbohydrates is not observed at 1080 cm^−1^.

### INH-stress condition

**Fig. 2:**
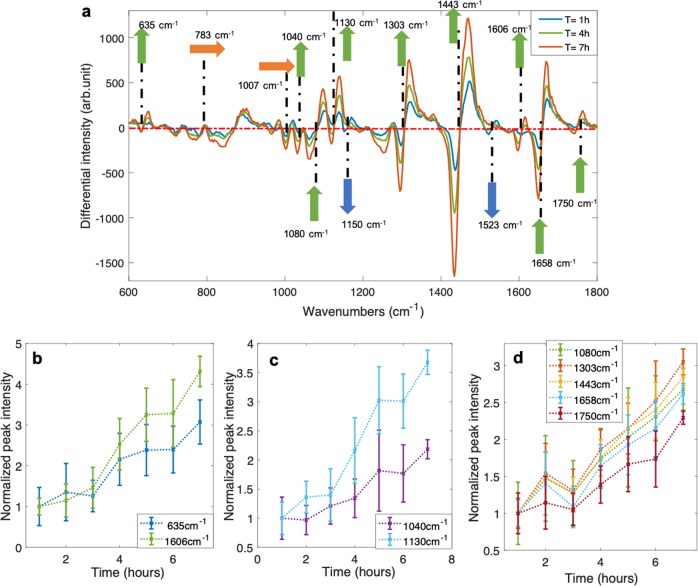
INH-stress experiment. **a** Shows the average WMR spectra for an INH-stress experiment, presenting the first (T = 1 h; blue spectrum), an intermediate (T = 4 h; green spectrum) and the last (T = 7 h; red spectrum) time point of the experiment. The green, orange and blue arrows are, respectively, showing the Raman peaks that are increasing, stable and decreasing over time based on the three INH-stress experiments. The *x*-axis presents the wavenumbers in cm^−1^, while the *y*-axis shows the intensity in arbitrary unit. In WMR spectra, the zero crossings on the red dash-dotted line shows the positions of the Raman peaks. **b**–**d** Present the evolution of Raman peak intensities over time. **b** Shows the Raman peaks at 635 cm^−1^ and 1606 cm^−1^ (associated with tyrosine) intensities during the experiment. **c** Presents the Raman peaks at 1040 cm^−1^ and 1130 cm^−1^ (associated with carbohydrates) intensities over time. **d** Shows the Raman peaks at 1080, 1303, 1443, 1658 and 1750 cm^−1^ (associated with lipids) intensities over the course of experiment. The *x*-axis shows time in hours, and *y*-axis the normalised intensity of the Raman peaks.

After investigating the behaviour of organisms in the "no-stress" system, we wanted to examine how they respond to stress: in this case, the antibiotic pressure. In the second group of experiments, a suspension of 7-day-old *M. smegmatis* was re-suspended in fresh 7H9 broth with INH at the minimum inhibitory concentration (MIC) level (see details on the MIC determination in Supplementary section S3.6 and Supplementary Fig. 9) and trapped. The bacteria were then measured with WMR spectroscopy for up to 8 h. Average Raman spectra, of one INH-stress experiment, corresponding to the first (T = 1 h), fourth (T = 4 h) and seventh (T = 7 h) hours are presented in Fig. 2a. Average Raman spectra for all INH-stress experiments are shown in Supplementary Fig. 2. The evolution of Raman peaks over time for all experiments are presented in Supplementary Figs. 3–8.

The main difference between INH-stress experiments and no-stress experiments is the increase over time of the intensity in Raman peaks located at 1080, 1303, 1443, 1658 and 1750 cm^−1^. These Raman peaks increase with a similar pattern over time, two (1303 and 1750 cm^−1^) are mainly associated with lipids (see Fig. 2a, d; Supplementary Fig. 5d–f). This suggests that the increase observed in these five Raman peaks is mainly driven by lipids. In contrast, the Raman peaks associated with lipids were stable or slightly decreasing in the no-stress condition (see Fig. 1a, d; Supplementary Fig. 5a–c). In all three INH-stress experiments, Raman peaks associated with lipids increased; only in the second biological repeats after a strong increase in the first 5 h, Raman peaks associated with lipids started to decrease fast. However, all Raman peaks were found to decrease (780, 1007, 1040, 1080, 1130, 1300, 1443, 1658 and 1750 cm^−1^) or stabilise (635 and 1606 cm^−1^) after *t* = 5 h, suggesting that an important change in the biological composition of the trapped bacteria occurred at that time point.

The Raman peak at 1007 cm^−1^, corresponding to phenylalanine, was found to be stable overall, similar to the no-stress condition, over the course of the experiments (see Supplementary Fig. 7d–f). The increase, associated with tyrosine, observed in Raman peaks at 635 cm^−1^ and 1606 cm^−1^, was reduced and delayed compared with the no-stress experiments (Fig. 1b and Fig. 2b).

The Raman peaks at 1040 cm^−1^ and 1130 cm^−1^ were also found to be increasing over time. But similar to the Raman peaks at 635 cm^−1^ and 1606 cm^−1^, the increase was reduced and delayed compared with the no-stress experiments (Fig. 1c and Fig. 2c).

Moreover, in all no-stress experiments, both Raman peaks at 1040 cm^−1^ and 1130 cm^−1^ showed a comparable trend, while in the INH-stress condition, in two experiments out of three, the Raman peak at 1130 cm^−1^ increases more than that at 1040 cm^−1^ (see Supplementary Fig. 4).

The Raman peak at 783 cm^−1^, corresponding to nucleic acids, was also found to be stable over time. This is different in comparison with the no-stress experiments where an increase at 783 cm^−1^ was observed over time (see Supplementary Figs. 2 and 8). The Raman peaks at 1150 cm^−1^ and 1523 cm^−1^, corresponding to carotenoids, were still decreased over time, and similar to the no-stress experiments, both peaks were almost not visible after 8 h of the measurement.

In order to understand whether the increase in the Raman peaks at 635, 1040, 1130 and 1606 cm^−1^ were due to aggregation, acoustic force or both, an experiment was designed as described in Supplementary section S3.7 and Supplementary Fig. 10. Bacteria were first trapped for 2 h, released for 2 h, then new bacteria were pushed in the chamber. These steps were repeated to trap and measure three groups of bacteria. Raman spectra were acquired during the first, fifth and ninth hour of each experiment. No increase over time in the Raman peaks at 635, 1040, 1130 and 1606 cm^−1^ were observed in those experiments (see Supplementary Tables 2 and 3). This suggests that the increase in those Raman peaks that was associated with tyrosine and carbohydrate was triggered by the acoustic force or the fact that bacteria were aggregated in close proximity.

## Discussion

The ability to target a given bacterial cell population and observe the effect of culture conditions and drugs on the cells in real time is an important development that will allow us to answer a number of complex questions in bacteriology. The system presented in this study integrates both Raman spectroscopy and acoustic trapping, and therefore permits us to make continuous measurement, using a non-destructive and label-free method over a period of many hours. The Raman spectra provide detailed qualitative and quantitative information of important chemical components of the bacteria cells, notably lipids and nucleic acids, based on their unique vibrational characteristics (fingerprints). These changes in the Raman fingerprints are extracted and interpreted to provide us an exquisitely sensitive measure of changes in the live cells. The acoustic trap suspends the cells in the surrounding media, and by holding them away from surfaces reduces Raman background signal, thus enhancing signal to noise ratio.

Our report shows that live bacterial population can be successfully trapped and their metabolic responses monitored over time in response to specific conditions. As well as being able to monitor the response to antibiotics, it could be used to monitor response to multiple changes (e.g.) pH, temperature, oxygen concentration, nutrient starvation and main carbon source. To understand better mycobacterial phenotypes and the conditions that favour lipid-rich cells, the accumulation of intracellular lipids and the conditions that induce the usage of lipids and lipid-poor cells, there is a need to develop a methodology that can follow the changes in live mycobacteria facing a specific condition in real time. Our combination of acoustic trapping and WMR spectroscopy enables us to follow in a quantitative and qualitative manner all the major components of the mycobacterial population over time.

In 2011, Wu et al. investigated lipids, using Raman spectroscopy, in microalgae cells and assigned the Raman peaks at 1075, 1300, 1440, 1650 and 1736 cm^−1^ to lipids^22^. In 2015, Stockel et al. studied mycobacterial species and assigned the peaks at 1081, 1305, 1446 and 1748 cm^−1^ to lipids^23^. In no-stress experiments, lipid-associated Raman peaks located at 1080, 1303, 1443, 1658 and 1750 cm^−1^ were found to be stable and or decreasing during the experiment, suggesting no important changes in lipid quantity in bacteria in this condition (Fig. 1a, d). Nucleic acids were increasing over time (Fig. 1).

INH is an antibiotic used in the current TB treatment regimen. The activation of INH by KatG, a mycobacterial enzyme, leads to the inhibition of nucleic acid and lipid synthesis^24^. This antibiotic is known to induce an inhibition of the synthesis of mycolic acids, a component of the cell wall of mycobacteria^25^. *M. smegmatis* INH MIC was 32 μg ml^−1^ in the conditions detailed in Supplementary Information section S3.6. The INH MIC of *M. smegmatis* in the chamber might be different, as the cell concentration is higher, the growth phase is different and the cells are aggregated and not planktonic. In all three INH-stress experiments, an increase in the lipid content in *M. smegmatis* cells was observed (see Fig. 2a, d; Supplementary Fig. 5d–f). A recent study, using mass spectrometry to investigate the effect of INH treatment on *M. tuberculosis* lipids, showed that INH treatment modified the composition in glycerolipids, glycerophospholipids and fatty acyls. A greater number of lipids were found in all lipid category in the INH-treated group^26^. They also observed a reduction in lipase activity in INH treated cells compared with the control. Their study, however, provided qualitative information only. We believe that our report is the first study showing, in real time, that INH induces an increase in lipid quantity in *M. smegmatis* cells. This observation raises concerns as a higher quantity in lipids in mycobacteria could render the cells more tolerant to antibiotics. Further work, investigating the effect of INH, should now focus on obtaining the quantitative data for specific lipid groups, such as triacylglycerols (TAGs). TAGs were previously shown to be an important component of intracellular lipophilic inclusions observed in mycobacteria^27^. This is important as the presence of bacteria with intracellular lipid bodies in patient’s sputum samples has been associated with poor long-term treatment outcome^19^. More generally, lipid-rich cells surviving treatment are thought to play a central role in patient relapse^21^. Further, in vitro and clinical studies exploring the role of lipid bodies in defining the outcome of infection and their use as biomarker for treatment outcome are still required^18^.

In the second INH-stress biological repeats, most Raman peaks were found to be reduced after *t* = 5 h, suggesting a reduction in most major cell components, see Supplementary Figs. 3–8. This observation could be explained by INH action that lead to cell death or lysis. This could be anticipated by the results of previous experiments where, after 6 h of exposure to 50 μg ml^−1^ of INH, *M. smegmatis* cells began to lyse^28^. INH can induce important structural changes on mycobacterial cells, such as alteration of cell poles leading to a release of material to the extracellular medium or cell deformation^29^.

Another clear effect of INH could be observed on the Raman peak located at 783 cm^−1^ (nucleic acids). This peak is stable over time in INH-stress experiments, while it was increasing over time in the no-stress condition in Supplementary Fig. 8. This observation is concurrent with previous work that showed that INH can inhibit nucleic acids synthesis in *M. tuberculosis*^30^.

Other changes in Raman spectra over time were clearly observed in both condition investigated and in all six experiments. Trapping planktonic bacteria induced an increase in the Raman peaks at 635 cm^−1^ and 1606 cm^−1^ associated with tyrosine and at 1040 cm^−1^ and 1130 cm^−1^ associated with carbohydrates. The increase in those Raman peaks is delayed and reduced in presence of INH as shown in Fig. 2 and in Supplementary Figs. 3 and 4. The production of both is related to the fact that bacteria are forced to aggregate due to the acoustic force as demonstrated in the experiments detailed in Supplementary section S3.7 and Supplementary Fig. 10. The production of tyrosine and carbohydrate seems, therefore, to be a response to either the aggregation or to the forces generated by the acoustic waves, as those two phenomena cannot be dissociated in this context. D-tyrosine has been previously linked with biofilms regulation^31^. In both Gram-positive and -negative bacteria, D-tyrosine, even at low concentrations, was shown to inhibit biofilm formation. In addition, D-tyrosine impacts the production of exopolysaccharides in bacterial species. However, the role of D-tyrosine on biofilm and exopolysaccharides production seems to be both concentration and species specific^31^. This suggests that more work on the production of tyrosine in *M. smegmatis* would be needed to fully understand what is happening here in this context. Similarly, the increase in carbohydrates observed in all trapping experiments requires further investigation. Further research is required to fully understand the effect that the acoustic trapping has on the bacterial population.

It was shown previously that INH impacts the composition in carbohydrates in mycobacteria^32,33^. This could explain why the Raman peak at 1130 cm^−1^ was increasing more over time than 1040 cm^−1^ in two out of three INH-stress experiments. Another explanation could be that the strong increase observed in lipids in the INH-stress condition could be also seen at 1130 cm^−1^ and not at 1040 cm^−1^.

In all six experiments, a clear reduction over time of the Raman peaks at 1150 and 1523 cm^−1^ associated with carotenoid was observed. After only few hours, these two Raman peaks are almost not visible. In addition, the initial concentration of carotenoids observed in the 7-day-old *M. smegmatis* culture was variable (see Supplementary Fig. 6).

## Methods

### Microfluidic chamber/acoustic trap system

**Fig. 3:**
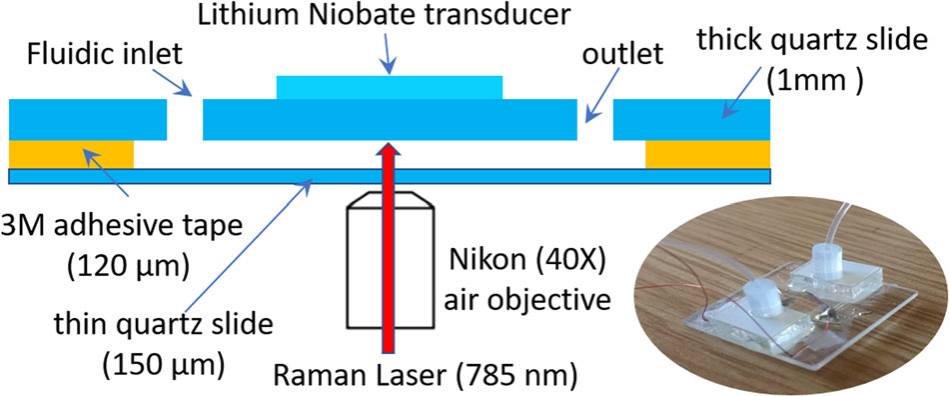
Design of acoustic trapping chamber (Indicative cross sectional view, not to scale). The inset shows a photo of the chamber.

A flow cell with acoustic trapping chamber, as shown in Fig. 3, was defined by a laser-cutting channel structure into double-sided transfer taper (3M 9629PC, creating a channel height of ~120 μm). This was bonded on one side by a 1-mm-thick quartz glass plate (25 ×  25 mm, SPI Supplies) which had two 1-mm holes drilled through it for fluidic inlet/outlet ports. A 150-μm-thick quartz coverslip formed the bottom surface (and acoustic reflector layer) of the device. The transparent transducer was formed from a 400-μm-thick piece of z-cut lithium-niobate (Roditi International Corporation Ltd) with 200-nm indium thin oxide (ITO) electrodes deposited in-house^34^. The back electrode was ‘wrapped around’ with silver conductive paint to a section of the front electrode that had been scored to electrically isolate it. Silver epoxy was used to make electrical connection to both terminals. The transducer was attached to the 1-mm quartz glass plate using epoxy (Epotek 301). Fluidic connections were made to the ports by attaching a laser-cut acrylic mounting plate (with double-sided transfer tape) to hold a short length of silicone tubing, into which PTFE (polytetrafluoroethylene) tubing was pushed with a friction fit. At the resonance frequency of 8.07 MHz (frequency determined by observation of test bead movement across a range of test frequencies), a half-wavelength standing wave is setup in the channel below the transducer, causing particles to be both levitated at the channel half-height (against gravity) and trapped in the lateral direction against flow. The acoustic pressure amplitude inside the capillary for a given drive voltage was estimated by balancing the weight of a 10-μm fluorescent polystyrene bead against the acoustic radiation force in the manner described by Spengler et al.^35^. Acoustic pressure was found to be related to the drive voltage applied to the transducer by a factor of 18.2 kPa/Vpp ± 30%.

Initial trapping of the bacteria (2–3 min) was at a pressure amplitude of 128 kPa (7 Vpp). This enabled formation of a thin layer of bacteria levitated in the centre of the channel. Once the aggregate formed, stabilised by secondary forces^13^, it was found possible to reduce the amplitude to 54.6 kPa (3 Vpp) thereafter, which helped reduce acoustic streaming. The time between the re-suspension in fresh broth and the first Raman spectrum was kept as short as possible (<5 min).

### Raman system

The Raman system was as described in our previous work, except that the fluorescence imaging function was not in use^9^. The confocal Raman system is based on a Nikon microscope (Nikon TE2000-E) using a tunable Ti:Sa laser (M2 SolsTis lasers, 1 W^@^785 nm) and an Andor spectrometer (Andor Shamrock SR303i). The acoustic chamber is placed on the stage of the microscope. About 100 mW of laser is applied at the sample plane to get strong enough signals without disturbing the acoustic trapping.

### Bacterial culture

*M. smegmatis* (NCTC 8159) was grown in Middlebrook 7H9 broth (Sigma-Aldrich) at 37 °C. The medium was supplemented with Tween 80 (0.05% (v/v), Fischer Scientific) and with 2 mL of glycerol for 450 mL of 7H9 broth.

### Sample preparation

All *M. smegmatis* cultures used were 7-day old. Bacterial concentration was investigated by plating serial dilution on 7H10 agar plates and calculating CFU ml^−1^ (see Supplementary section S3.2 and Supplementary Table 1). In total, 4 mL of culture were then harvested and spun down at 20,000 × g for 3 min, the supernatants were discarded, and the pellets were then re-suspended in 500 μl of fresh 7H9 medium (pre-warmed to 37 °C). In INH experiments, bacterial pellets were re-suspended in 500 μl of fresh 7H9 broth with isoniazid at 32 μg ml^−1^ (pre-warmed to 37 °C). Once re-suspended, the bacterial suspension was inserted into the microfluidic system, and pumped by a precision syringe pump (AL2000, World Precision Instruments). Bacteria were then trapped in the chamber using acoustic waves.

### WMR spectroscopy

Similar to our previous work^9,36–38^, five WMR spectra were recorded with, in this work, a 50 s integration time for each spectrum from acoustically trapped bacteria. During acquisition, the laser line was tuned over a total modulation range of 1.5 nm with five steps. A single WMR spectrum can be reconstructed from these five spectra with all background fluorescence being removed essentially. As a differential spectrum, the WMR spectrum had zero crossings corresponding to the Raman peaks in the traditional Raman spectrum while their peak intensity will be indicated by the peak-to-valley value near the zero crossing.

### Experimental parameters

Experimental parameters such as temperature, bacterial concentration, laser power, acoustic amplitude and frequency were optimised as described in Supplementary section S3.1 and Supplementary Fig. 1. These values were used unless otherwise stated: the acoustic amplitude was set at 55 kPa (3 Vpp) during the experiment and the frequency at 8.07 MHz. The bacterial concentration, inside the chamber, for all the experiments is shown in Supplementary section S3.2 and ranged from 3.1 × 10^8^ CFU ml^−1^ to 1.0 × 10^9^ CFU ml^−1^. The laser power was controlled throughout the experiment to ensure that the power remained close the initial value set at t = 0 h, always close to 100 mW. The laser power for all experiments and time points are shown in Supplementary section S3.3. The temperatures were close to 37 °C and recorded, throughout the experiments, using sensors attached on the surface of the chamber. The temperature values, for all experiments, are presented in Supplementary section S3.3. The acquisition time for each WMR spectrum was set to 250 s in total per spectrum.

### Contamination control

The 7-day-old *M. smegmatis* cultures used were first controlled for purity on brain heart infusion (BHI) agar plates. The bacterial suspensions were also controlled for purity after the experiment and plated on BHI agar. Between experiments, the chamber was cleaned and incubated with a Virkon solution and rinsed using phosphate-buffered saline (PBS).

### WMR spectra analysis

The analysis of Raman spectra focused in the fingerprint region between 600 cm^−1^ and 1800 cm^−1^.

### Reporting summary

Further information on research design is available in the Nature Research Reporting Summary linked to this article.

## Supplementary information

Supplementary Information

Reporting Summary

## Data Availability

The data that support the findings of this study are available from the corresponding authors upon reasonable request or directly available online^39^.

## Code availability

The software codes used for analysing the data are available from the corresponding authors upon reasonable request.
