# Supplementary material for: Real-time monitoring of live mycobacteria with a microfluidic acoustic-Raman platform
Source: Commun Biol. 2020 May 14;3:236. doi: 10.1038/s42003-020-0915-3 (PMC7224385; doi:10.1038/s42003-020-0915-3)
Supplement: Supplementary file 1 — Supplementary Information [file 42003_2020_915_MOESM1_ESM.pdf]

Supplementary Information: Real-time monitoring of live mycobacteria with a microfluidic acoustic-Raman platform

S1 Supplementary Figures

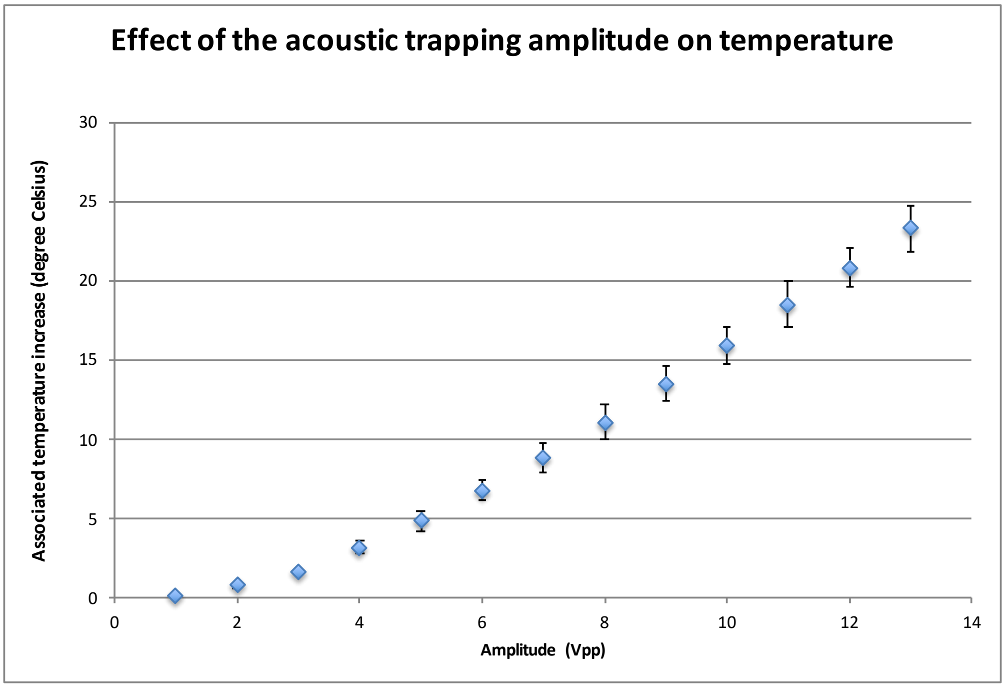

Supplementary Figure 1: Effect of the acoustic trapping amplitude on temperature. The error bar represents ±1 standard deviation. The x-axis shows the amplitude in Vpp and the y-axis presents the associated temperature increase in degree Celsius.

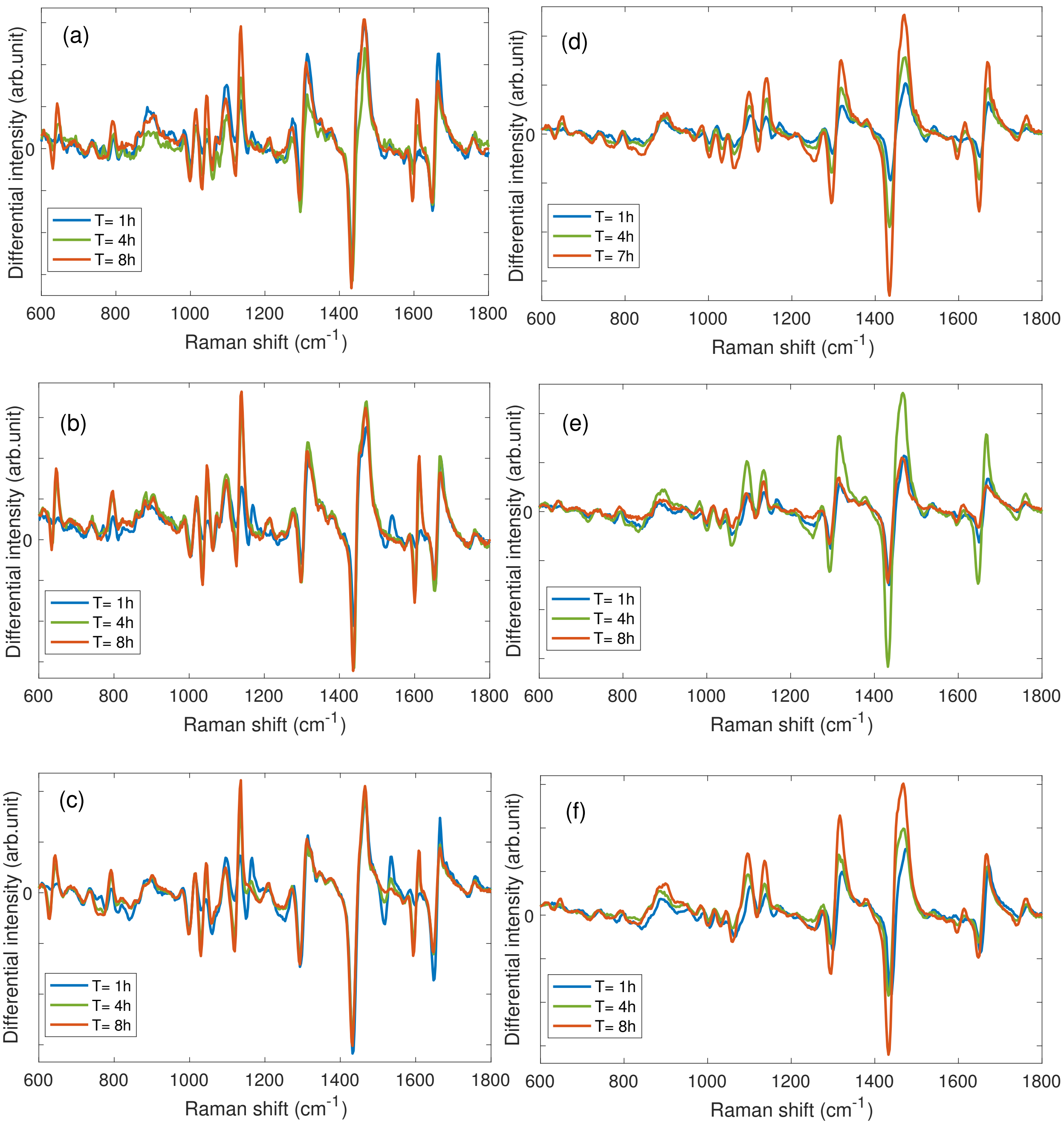

Supplementary Figure 2: Averaged WMR spectra for no-stress experiment (a – c) and the INH-stress experiments (d – f) at T = 1h , 4h and 7 or 8h.

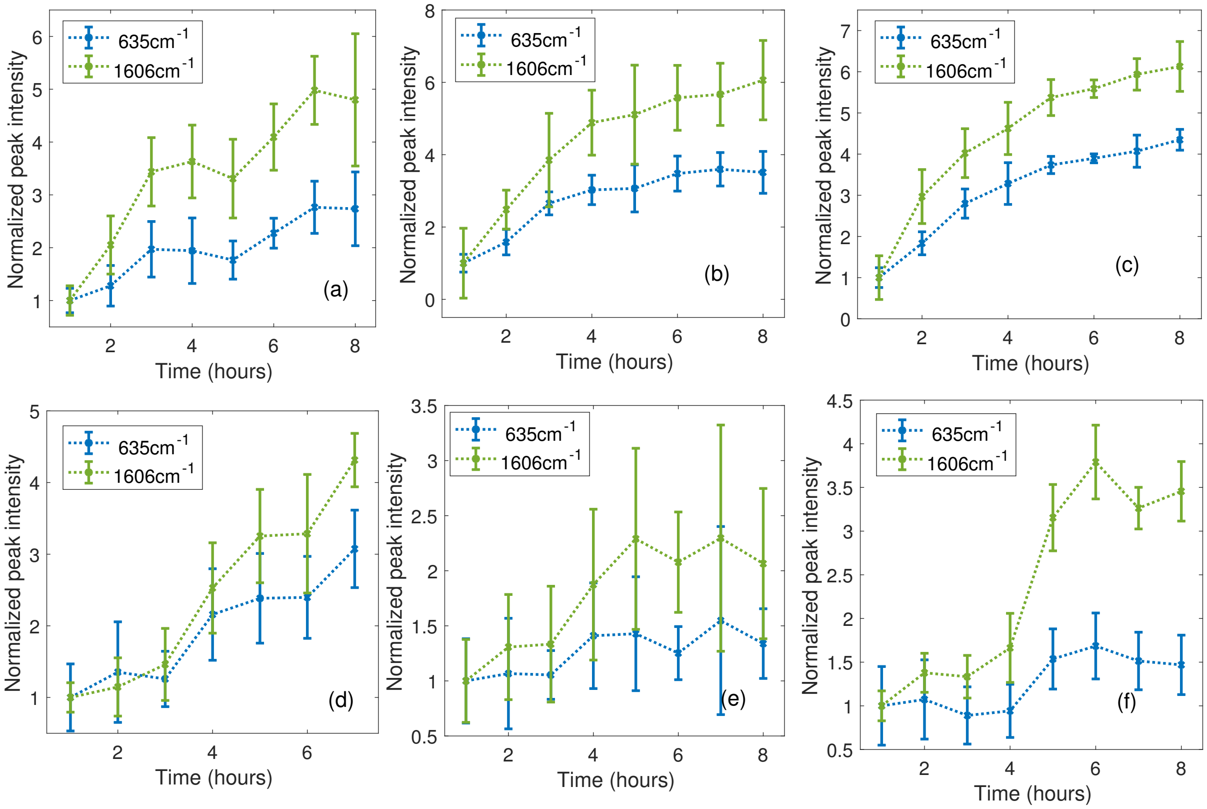

Supplementary Figure 3: Evolution of Raman peaks at 635 cm^{-1} and 1606 cm^{-1} over time. Figures (a – c) correspond to changes in both Raman peaks in the three no-stress experiments while figures (d – f) represent the changes in the same Raman peaks in the three INH-stress experiments. The x-axis represents the time in hours and the y-axis displays the normalized peak intensity.

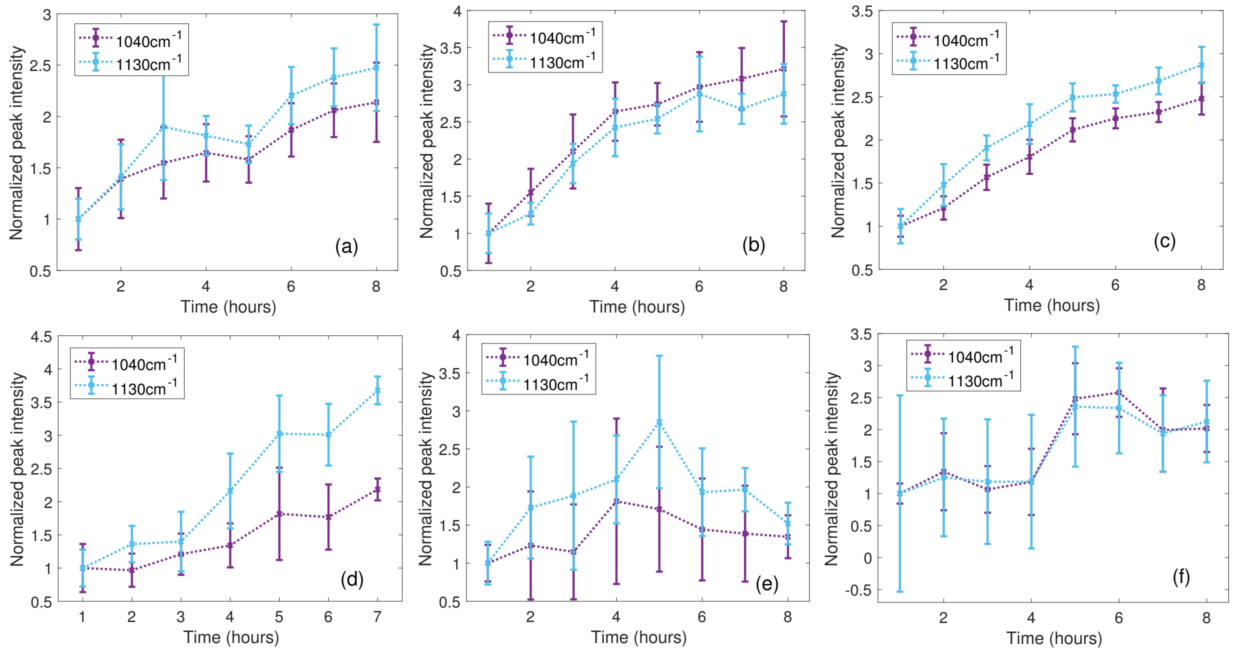

Supplementary Figure 4: Evolution over time of the Raman peak intensity at 1040 cm^{-1} and 1130 cm^{-1}. Figures (a – c) correspond to changes in both Raman peaks in the three no-stress experiments while figures (d – f) represent the changes in the same Raman peaks in the three INH-stress experiments. The x-axis represents the time in hours and the y-axis displays the normalized peak intensity.

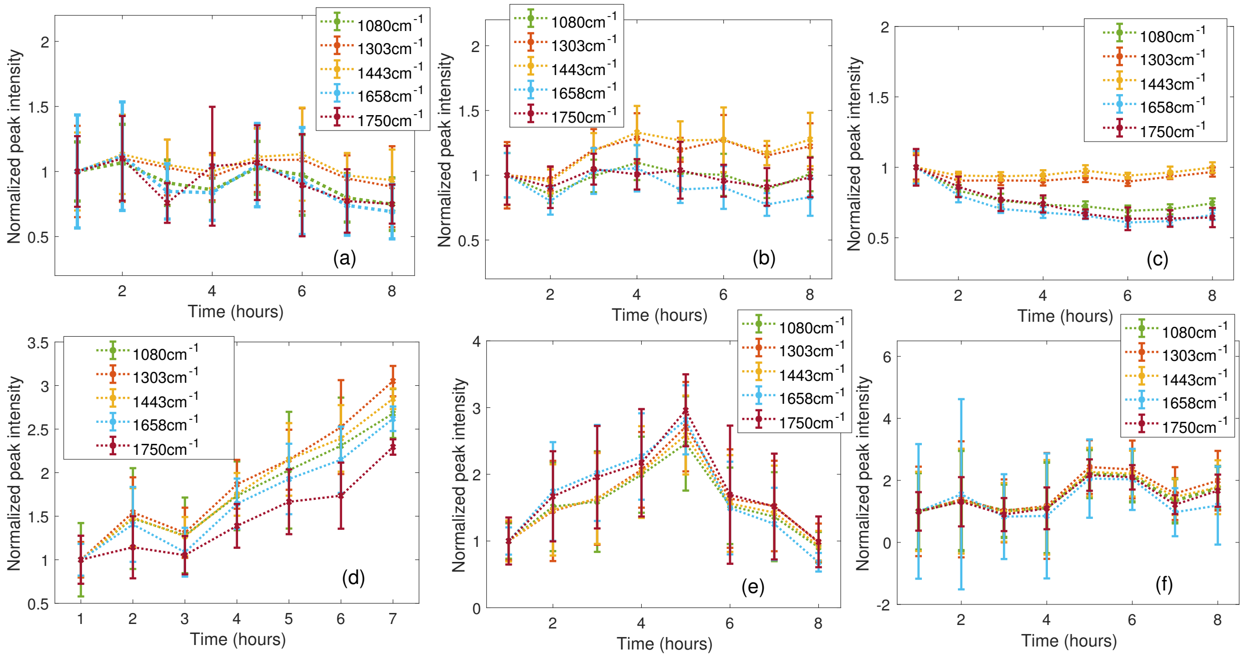

Supplementary Figure 5: Evolution of the Raman peak intensity at 1080 cm^{-1}, 1300 cm^{-1},1443 cm^{-1}, 1658 cm^{-1}and 1750 cm^{-1} over time. Figures (a – c) correspond to changes in both Raman peaks in the three no-stress experiments while figures (d – f) represent the changes in the same Raman peaks in the three INH-stress experiments. The x-axis represents the time in hours and the y-axis displays the normalized peak intensity.

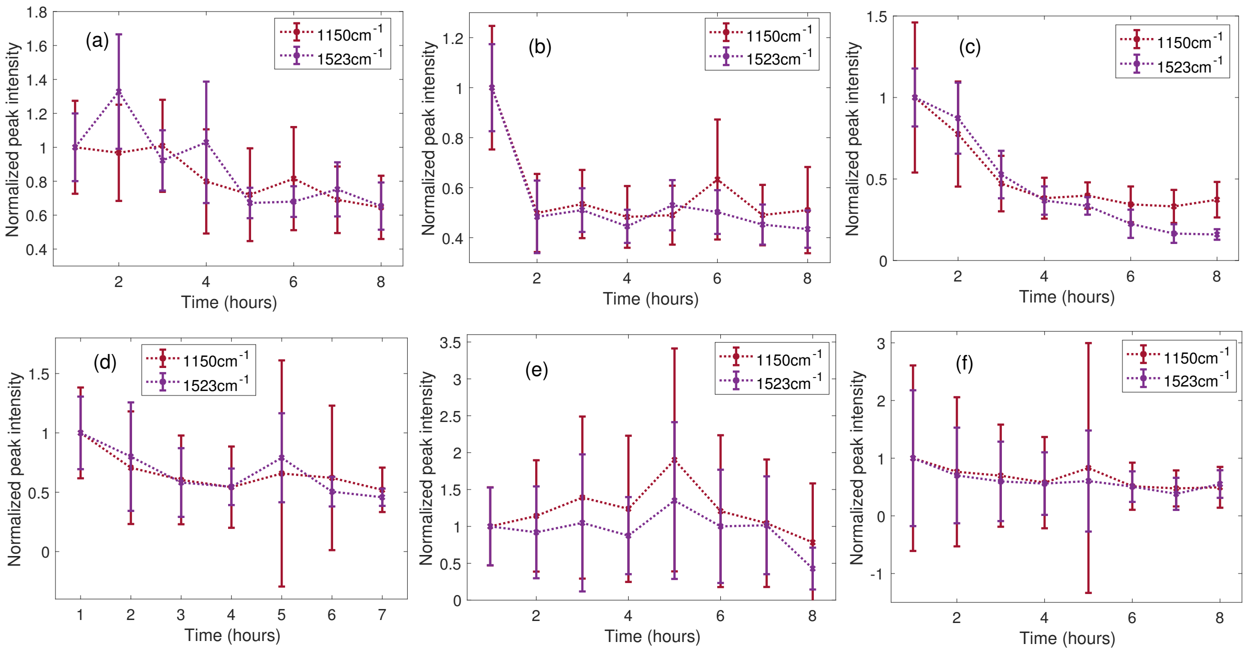

Supplementary Figure 6: Evolution of the Raman peak intensity at 1150 cm^{-1} and 1523 cm^{-1} over time. Figures (a – c) correspond to changes in both Raman peaks in the three no-stress experiments while figures (d – f) represent the changes in the same Raman peaks in the three INH-stress experiments. The x-axis represents the time in hours and the y-axis displays the normalized peak intensity.

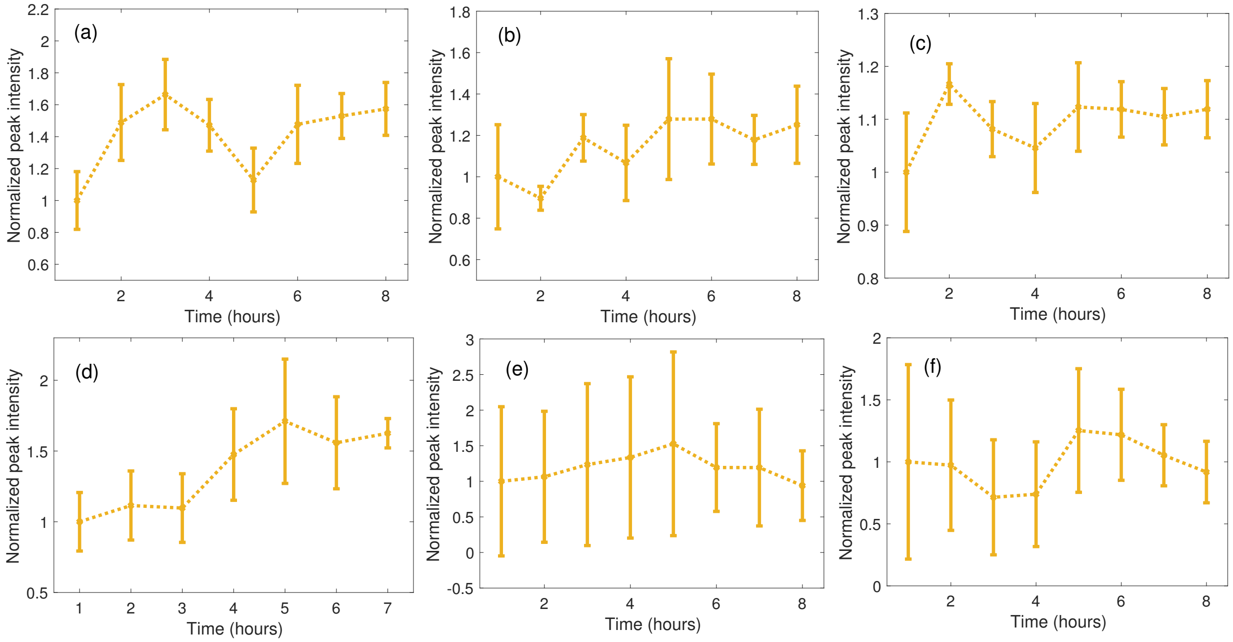

Supplementary Figure 7: Evolution of the Raman peak intensity at 1007 cm^{-1} over time. Figures (a – c) correspond to changes in both Raman peaks in the three no-stress experiments while figures (d – f) represent the changes in the same Raman peaks in the three INH-stress experiments. The x-axis represents the time in hours and the y-axis displays the normalized peak intensity.

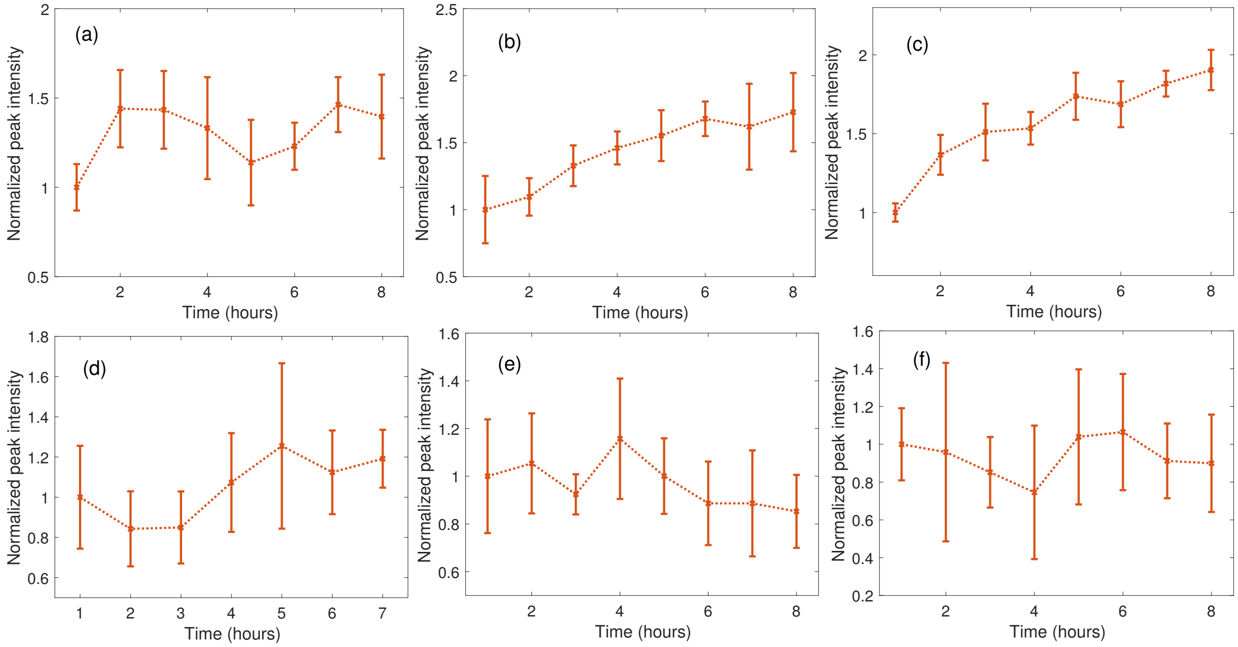

Supplementary Figure 8: Evolution of the Raman peak intensity at 783 cm^{-1} over time. Figures (a – c) correspond to changes in both Raman peaks in the three no-stress experiments while figures (d – f) represent the changes in the same Raman peaks in the three INH-stress experiments. The x-axis represents the time in hours and the y-axis displays the normalized peak intensity.

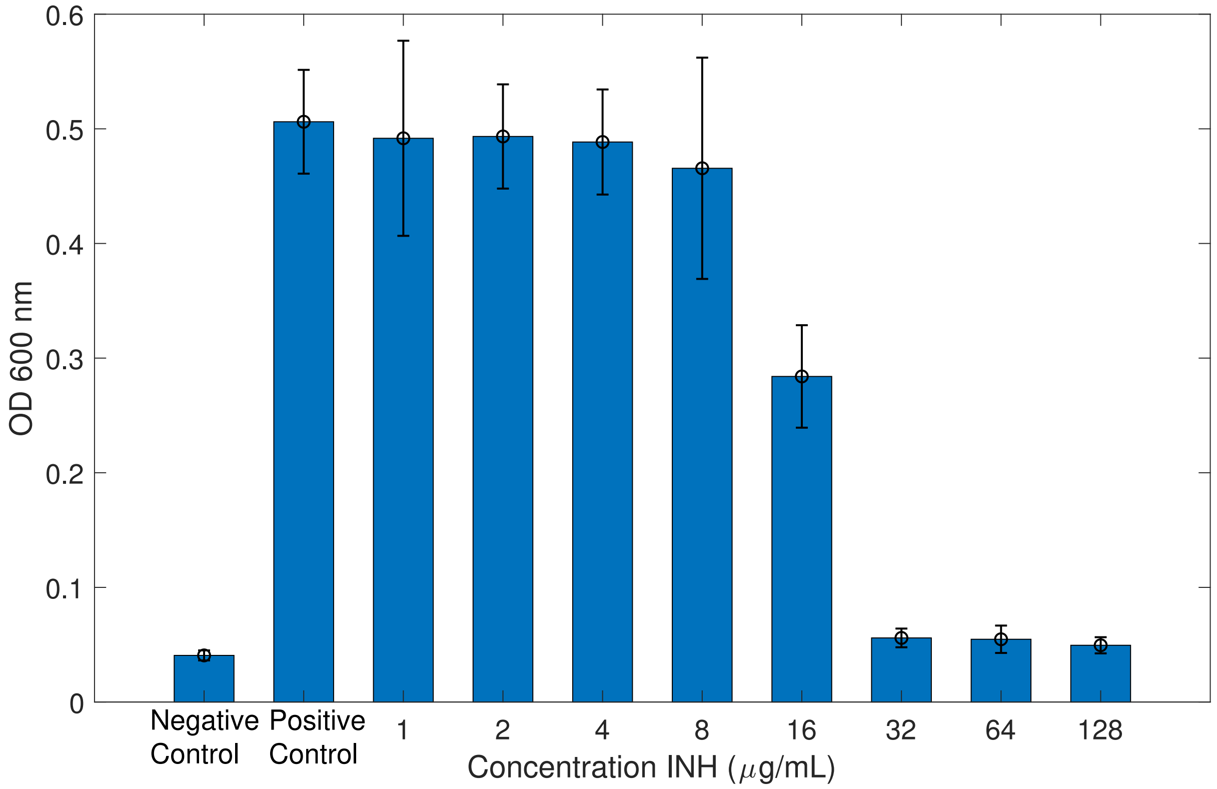

Supplementary Figure 9: *M. smegmatis* against doubling dilutions of INH. Four biological replicates were made with technical triplicate experiments yielding an n = 12 for each condition tested. Error bars represent 1 standard deviations.

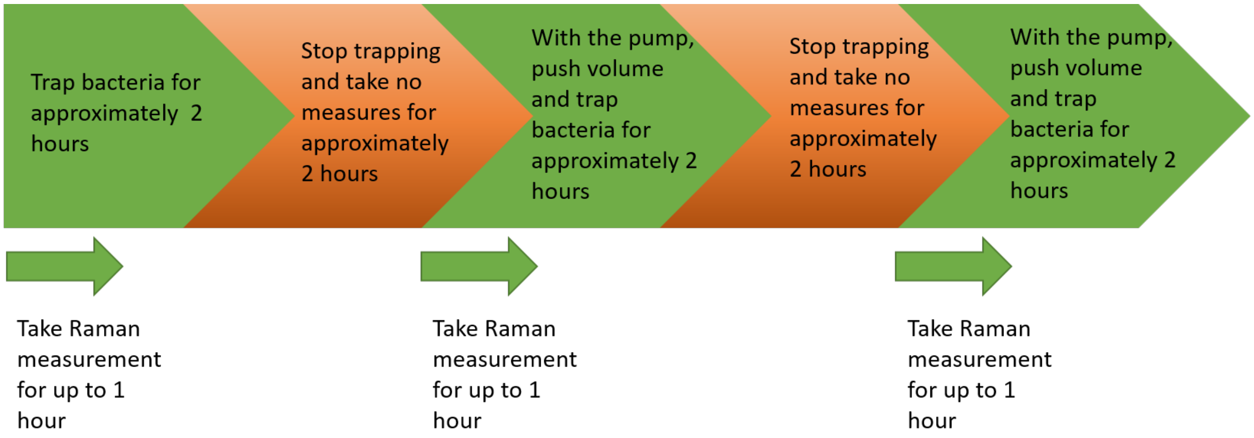

Supplementary Figure 10: Experimental flow plan.

S2 Supplementary Tables

Experiments	CFU·ml^{-1} in initial culture	CFU·ml^{-1} (estimated) in the acoustic chamber
no-stress experiment 1	1.1×10^8	8.8×10^8
no-stress experiment 2	9.2×10^7	7.4×10^8
no-stress experiment 3	7.5×10^7	6.0×10^8
INH-stress experiment 1	1.3×10^8	1.0×10^9
INH-stress experiment 2	5.1×10^7	4.1×10^8
INH-stress experiment 3	3.9×10^7	3.1×10^8

Supplementary Table 1: Bacterial concentration in different experiments.

Raman shift	Peak intensity (1st hour)	Peak intensity (5th hour)	Peak intensity (9th hour)
635 cm^{-1}	1.00±0.41	1.14±0.34	0.99±0.36
1040 cm^{-1}	1.00±0.22	1.15±0.24	0.89±0.20
1130 cm^{-1}	1.00±0.30	1.26±0.28	1.05±0.36
1606 cm^{-1}	1.00±0.32	1.51±0.36	1.18±0.30

Supplementary Table 2: Peak intensities normalized by peaks from the first hour to show that there is no continuous increase of those Raman peaks over time in experiment one.

Raman shift	Peak intensity (1st hour)	Peak intensity (5th hour)	Peak intensity (9th hour)
635 cm^{-1}	1.00±0.27	1.16±0.41	1.12±0.33
1040 cm^{-1}	1.00±0.29	1.23±0.34	0.95±0.20
1130 cm^{-1}	1.00±0.47	1.34±0.70	1.12±0.21
1606 cm^{-1}	1.00±0.27	1.09±0.30	1.18±0.63

Supplementary Table 3: Peak intensities normalized by peaks from the first hour to show that there is no continuous increase of those Raman peaks over time in experiment two.

S3 Supplementary Notes

S3.1 Optimisation of the experimental parameters

The experimental parameters were first optimised to obtain a stable trap, a good Raman signal compared to the noise level and also a controlled temperature. A plastic cover was placed on top of the system and permits to control the temperature inside. The temperature had to be close to 37°C for the experiments as it is the optimal growth temperature for *M. smegmatis*. First, we investigated the impact of the amplitude of the acoustic trapping on the temperature. The temperature was set at 35.5°C inside the system and measured by sensors located on top of the chamber. Supplementary Fig. 1 shows the relationship between the amplitude in Vpp and the temperature in degree Celsius. It was important to use the lowest Vpp possible to minimise the potential impact of the trapping force on bacteria. With 5 Vpp we observe already an increase of 4.85 ± 0.65 (1 standard deviation, SD)°C; we validated 3 Vpp that still produce a stable trap and only increase the temperature by 1.65 ± 0.19 (1 SD)°C.

The bacterial concentration also had to be high as more bacteria facilitate and reinforced the trapping. Stable trap was achieved when using 4 mL of 7-day-old *M. smegmatis* re-suspended, after centrifugation and removal of the supernatants, in 500 µl of medium (8 times concentrated).

The bacteria were first trapped using 7 Vpp for 2 to 3 minutes, generating large aggregates of bacteria then the amplitude was reduced to 3 Vpp to maintain the trap throughout the experiment and the Raman measures. This helped creating a stable trap as once the bacteria were aggregated a lower trapping force was needed to maintain the trap.

The laser power had to be optimised to not disturb the trapping. With a laser power of 100 mW targeting trapped cells as described above we observed that the laser force was not breaking the trap and permitted to acquire wavelength modulated Raman (WMR) spectroscopy measures. The acquisition time was optimised using those conditions to obtain good quality Raman spectra

with good signal to noise ratio. 50 seconds per spectrum of acquisition time was validated and used for all the experiments presented. The laser itself induced a small temperature increase of about 0.5 °C.

S3.2 Bacterial concentration in the experiments

The bacterial concentration, in the chamber, for all the experiment is show in Supplementary Table 1 and ranged from 3.1×10^{8} CFU$\cdot$ ml^{-1} to 1.0×10^{9} CFU$\cdot$ ml^{-1}. The CFU$\cdot$ ml^{-1} were calculated using the 7-day-old cultures. Then 4 ml were spun down at 20,000 $\times$g for 3 minutes, the supernatants were discarded, and the pellets were then re-suspended in 500 µl of medium leading to 8 times concentrated bacterial suspensions.

S3.3 Temperature and laser power during the experiments

The laser power was controlled throughout all the experiments to ensure that the power remain close the initial value set at T = 0h always close to 100 mW. The laser power and the temperature in the chamber were monitored, every hour, for all experiments and time points. In the first no-stress experiment, on average the laser power was at 112.01 $\pm$ 1.93 (1 SD) mW and the temperature at 35.94 $\pm$ 0.46 (1 SD) °C. In the second no-stress experiment, on average the laser power was at 97.46 $\pm$ 3.48 (1 SD) mW and the temperature at 36.37 $\pm$ 0.29 (1 SD) °C. In the third no-stress experiment, onaverage the laser power was at 107.83 $\pm$ 3.67 (1 SD) mW and the temperature at 36.23 $\pm$ 0.31 (1 SD) °C. In the first INH-stress experiment, on average the laser power was at 100.56 $\pm$ 2.83 (1 SD) mW and the temperature at 36.19 $\pm$ 0.41 (1 SD) °C. In the second INH-stress experiment, on average the laser power was at 103.67 $\pm$ 4.09 (1 SD) mW and the temperature at 36.26 $\pm$ 0.22 (1 SD) °C. In the third INH-stress experiment, on average the laser power was at 108.63 $\pm$ 8.07 (1 SD) mW and the temperature at 36.14 $\pm$ 0.69 (1 SD) °C.

S3.4 Comparison of all biological replicates averaged Raman spectra

In the no-stress experiments a suspension of 7-day-old *M. smegmatis* was re-suspended in fresh 7H9 broth and trapped acoustically in the chamber. In the INH-stress experiments a suspension of 7-day-old *M. smegmatis* was re-suspended in fresh 7H9 broth with isoniazid (INH) at 32 $\mu g \cdot ml^{-1}$ and acoustically trapped in the same chamber. The bacteria were then measured using WMR spectroscopy for up to 8 hours during which both the temperature and the laser power were controlled every hour. The analysis of spectra focused in the fingerprint region between 600 cm^{-1} and 1800 $^{-1}$. Averaged WMR spectra acquired from three replicates are shown in Supplementary Fig. 2 (a – c) for the no-stress experiment and Supplementary Fig. 2 (d – f) for the INH-stress condition. The Raman peaks positions and their assignment are shown in Table 1. For clarity, only the averaged WMR spectra at the first (T = 1h), an intermediate (T = 4h) and the last (T = 7h or 8h) time points are presented in Supplementary Fig. 2.

S3.5 Evolution of Raman peaks over time in no-stress and INH-stress conditions

Raman peaks at 635 cm^{-1} and 1606 cm^{-1}. Supplementary Fig. 3 presents the evolution over time of the intensity of the Raman peaks located at 635 cm^{-1} and 1606 cm^{-1}. The increase in those Raman peaks was associated with tyrosine (See Table 1). In all 6 experiments we observe an increase in intensity in those two Raman peaks. However, in the INH-stress condition the increase is less strong and delayed in time. In the second INH-stress experiment, (Supplementary Fig. 3 (e)) the intensity of the Raman peaks stabilises after 5 hours of measurement. In the third INH-stress experiment, we noted that in Supplementary Fig. 3 (f) the intensity of the Raman peaks at 635 cm^{-1} is not showing a matching pattern with 1606 cm^{-1} which was the case in all other experiments.

Raman peaks at 1040 cm^{-1} and 1130 cm^{-1}. Supplementary Fig. 4 presents the evolution over time of the intensity of the Raman peaks at 1040 cm^{-1} and 1130 cm^{-1}. The increase in the these

two Raman peaks were mainly associated with carbohydrate; it is also possible that 1130 cm^{-1} shows some information related to lipids in the INH-stress condition (See Table 1). In all six experiments we observe an increase in both Raman peaks. However, in the INH-stress condition the increase is less strong and delayed. In the second INH-stress experiment, (Supplementary Fig. 4 (e)) the intensity of the Raman peaks reduces after five hours of measurement.

Raman peaks at 1080 cm^{-1}, 1300 cm^{-1}, 1443 cm^{-1}, 1658 cm^{-1} and 1750 cm^{-1}. Supplementary Fig. 5 presents the evolution over time of the intensity of the Raman peaks located at 1080 cm^{-1}, 1300 cm^{-1}, 1443 cm^{-1}, 1658 cm^{-1} and 1750 cm^{-1} associated mainly with lipids or lipids and proteins. The increase observed in those Raman peaks in the INH-stress condition were associated with Lipids (See Table 1). In the three no-stress experiments we observed that the intensity of those Raman peaks is generally stable over time. The intensity of several Raman peaks, 1080 cm^{-1}, 1658 cm^{-1} and 1750 cm^{-1} is, on overall, slightly reducing over time. In contrast, in the INH-stress conditions the intensity of the Raman peaks at 1080 cm^{-1}, 1300 cm^{-1}, 1443 cm^{-1}, 1658 cm^{-1} and 1750 cm^{-1} is strongly increasing with the a comparable trend for all peaks. In the second INH-stress experiment, we noted that in Supplementary Fig. 5 (e) the intensity of all those Raman peaks is rapidly reducing after 5 hours of measurement.

Raman peaks at 1150 cm^{-1} and 1523 cm^{-1}. Supplementary Fig. 6 presents the evolution over time of the intensity of Raman peaks at 1150 cm^{-1} and 1523 cm^{-1} associated with carotenoids (See in Table 1). In the three no-stress experiments we observe that the intensity of those two Raman peaks is rapidly decreasing over time. In the INH-stress experiments the intensity of the Raman peak at 1150 cm^{-1} and 1523 cm^{-1}, is also reducing over time. In the second INH-stress experiment, Supplementary Fig. 6 (e), the intensity of the Raman peaks begin to reduce after five hours of measurement.

Raman peak at 1007 cm^{-1}. Supplementary Fig. 7 presents the evolution over time of the intensity of the Raman peak located at 1007 cm^{-1} associated with phenyalanine (See Table 1). In no-stress and INH-stress experiments, we observe that the intensity of this Raman peak is relatively stable over time; only in Supplementary Fig. 7 (d) a small increase is observed.

Raman peak at 783 cm^{-1}. Supplementary Fig. 8 presents the evolution over time of the intensity of the Raman peak at 783 cm^{-1} associated with nucleic acids (See Table 1). In the no-stress condition the intensity of the Raman peak at 783 cm^{-1} is slightly increasing over time. In INH-stress condition, we observe that the intensity of the Raman peak at 783 cm^{-1} is relatively stable over time with the exception of the second INH-stress experiment, Supplementary Fig. 8(e), in which the intensity of this Raman peak is reducing after 4 hours of measurement.

S3.6 INH MIC

M. smegmatis (NCTC 8159) was grown from glycerol stock for 3 days in Middlebrook 7H9 (Sigma Aldrich, UK) and on Middlebrook 7H11 (Sigma Aldrich, UK) until the liquid culture read 0.01 OD600. This culture was used thereafter with no further sub-culturing steps. Doubling dilutions of INH (Sigma Aldrich, UK) were produced from 1280 $\mu g \cdot ml^{-1}$ to 10 $\mu g \cdot ml^{-1}$ (8x serial dilutions). 20 µl of each of these solutions were aliquoted into the wells of a sterile 96-well plate (Nunc, DK) in triplicate along with 6 wells containing sterile water (negative & positive controls) for a total of 30 wells. 160 µl of sterile Middlebrook 7H9 media was added to each of the 30 wells. 20 µl of *M. smegmatis* culture was then aliquoted into 27 of the wells (with the exception of the negative control wells) for a final concentration estimated to be 5x105 cfu/mL. The negative control wells then had another 20 µl of sterile Middlebrook 7H9 added. With all the components added the total volume of each well was 200 µl and the INH added had been diluted 10x making the final concentration range seen in Supplementary Fig. 9. A 1:10 dilution of the original *M. smegmatis* stock was then further diluted 8x serially and plated onto a Middlebrook 7H11 agar plate for cfu counting in the Miles and Misra fashion. This was to confirm the cfu estimate made previously.

After 3-5 days, or until confluent growth was evident in the positive control wells and no growth was seen in the negative control wells, the 96-well plate was removed from the incubator. The plate was then analysed in a 96-well plate reader at 600nm absorbance. These steps were repeated 4 times to yield a total n number of 12 for each dilution of INH. Supplementary Fig. 9 shows the dose dependant relationship between INH and *M. smegmatis*. The MIC was found to be between 16 and 32 $\mu g \cdot ml^{-1}$ with 32 $\mu g \cdot ml^{-1}$ yielding the MIC90 and 16 $\mu g \cdot ml^{-1}$ yielding the MIC50.

S3.7 Discontinued measurement

In order to understand if the strong increase, observed in all previous experiments, of the Raman peaks at 635 cm^{-1}, 1040 cm^{-1}, 1130 cm^{-1} and 1606 cm^{-1} was associated with the trapping itself (either a reaction to the Acoustic force or a reaction to the cell aggregation) an experiment using discontinuous measurement was designed as shown in Supplementary Fig. 10.

The bacterial concentration, in the chamber, was 3×10^8 CFU·ml^{-1} for the first experiment and 2.8×10^8 CFU·ml^{-1} for the second. The temperature and laser power are well controlled during the two repeated experiments. No continuous increase of those Raman peaks is observed over time in both experiments (see Supplementary Table 2 and 3), suggesting that the increase in intensity over time observed in the experiment using continuous acoustic trapping is associated with the cell aggregation or is a reaction to the acoustic force.
